# Circulating bioactive sclerostin levels in an Austrian population-based cohort

**DOI:** 10.1007/s00508-021-01815-0

**Published:** 2021-02-05

**Authors:** Katharina Kerschan-Schindl, Ursula Föger-Samwald, Andreas Gleiss, Stefan Kudlacek, Jacqueline Wallwitz, Peter Pietschmann

**Affiliations:** 1 Department of Physical Medicine, Rehabilitation and Occupational Therapy, Medical University of Vienna, Waehringer Guertel 18–20, 1090 Vienna, Austria; 2 Department of Pathophysiology and Allergy Research, Center of Pathophysiology, Infectiology and Immunology, Medical University of Vienna, Vienna, Austria; 3 Center of Medical Statistics, Informatics, and Intelligent Systems, Medical University of Vienna, Vienna, Austria; 4 Medizinische Abteilung, Krankenhaus Barmherzige Brüder, Vienna, Austria; 5 Department Pharmacology, Physiology and Microbiology, Division Pharmacology, Karl Landsteiner Privatuniversität für Gesundheitswissenschaften, Krems, Austria

**Keywords:** Gender, Age, Body mass index, Bone turnover markers, Bone mineral density

## Abstract

**Background:**

Circulating serum sclerostin levels are supposed to give a good estimation of the levels of this negative regulator of bone mass within bone. Most studies evaluating total serum sclerostin found different levels in males compared to females and in older compared to younger subjects. Besides an ELISA detecting total sclerostin an ELISA determining bioactive sclerostin has been developed. The aim of this study was to investigate serum levels of bioactive sclerostin in an Austrian population-based cohort.

**Methods:**

We conducted a cross-sectional observational study in 235 healthy subjects. Using the bioactive ELISA assay (Biomedica) bioactive sclerostin levels were evaluated.

**Results:**

Serum levels of bioactive sclerostin were higher in men than in women (24%). The levels correlated positively with age (r = 0.47). A positive correlation could also be detected with body mass index and bone mineral density.

**Conclusion:**

Using the ELISA detecting bioactive sclerostin our results are consistent with data in the literature obtained by different sclerostin assays. The determination of sclerostin concentrations in peripheral blood thus appears to be a robust parameter of bone metabolism.

## Introduction

Diseases associated with high bone mass caused by mutation or deletion of the *SOST* gene led to the recognition of sclerostin [1, 2]. The glycoprotein sclerostin consisting of about 200 amino acids is mainly expressed by osteocytes. As an antagonist of the osteoanabolic Wingless-type mouse mammary tumour virus integration site (Wnt) pathway sclerostin is an important regulator of bone metabolism. Binding to and thus inactivating the lipoprotein receptor-related protein 5 and 6 (LRP‑5 and LRP-6) [3] leads to reduced osteoblast differentiation and activity. Thus, sclerostin inhibition was detected as a promising treatment option to preserve bone mass.

Circulating levels of sclerostin are supposed to reflect sclerostin levels in bone [4, 5]. Almost all investigations found higher sclerostin levels in males than in females [6–9]. Despite a few contradicting data [9, 10], most clinical studies detected an age-associated increase of serum levels of sclerostin [6, 11–14]. Although other companies also developed an assay, the majority of these studies used the quantitative sandwich enzyme-linked immunosorbent assay (ELISA) by Biomedica; however, by now the company has developed a new, a bioactive sclerostin ELISA. The previous assay used a polyclonal goat antibody and a monoclonal mouse antibody detecting various fragments of sclerostin proteins. The new assay is different. A recombinant monoclonal antibody binds to the second loop of the sclerostin core region capturing all sclerostin protein, which is able to bind to the LRP 5/6 complex of the Wnt signaling pathway thereby inhibiting bone formation. Thus, this ELISA captures all circulating proteins containing the free-receptor binding site, the bioactive sclerostin. The previous sclerostin ELISA as well as the bioactive sclerostin ELISA have been rigorously validated for clinical samples according to the Food and Drug Administration (FDA), the International Council for Harmonisation of Technical Requirements for Pharmaceuticals for Human Use (ICH), and the European Medicines Agency (EMA) guidelines. The bioactive sclerostin ELISA has been shown to correlate with other sclerostin ELISAs, including the one previously developed by Biomedica. But there are some hints that in different types of diseases the bioactive sclerostin ELISA shows different results than the commonly used ELISAs [15]. Nevertheless, only two investigations used the bioactive sclerostin ELISA so far. Patients with spinal cord injuries [16] as well as patients with alcoholic liver disease [17] had lower levels than healthy controls. Thus, the aim of this cross-sectional study was to investigate serum levels of the physiologically relevant sclerostin, the bioactive sclerostin in a population-based cohort and to compare our data with data from the literature using different ELISAs. This study had explorative character. The working hypothesis was that bioactive sclerostin ELISA shows similar results as previous studies using a sclerostin ELISA.

## Material and methods

### Participants

All subjects included in this study were participants of a previously published investigation [18]. The former population register-based study evaluated several biochemical parameters and bone mineral density (BMD). A total of 235 subjects studied at 4 different Austrian outpatient bone clinics were randomly selected for this study. All subjects were healthy and did not take any medication affecting bone metabolism. Women who were on hormone replacement therapy (HRT) within the previous 5 years were excluded as well. The study protocol was approved by the ethics committee of the Krankenhaus Barmherzige Brüder, Vienna and all participants provided written informed consent after the procedure of the trial had been explained to them.

### Study procedures

In all subjects, medical history was obtained and physical examination was performed. Their height was measured with a stadiometer and their weight was determined using a weight scale with a precision of 0.1 kg. Body mass index (BMI) was calculated as weight (kg) divided by the square of height (m). After overnight fasting blood samples were collected in sterile chilled tubes by standard venepuncture technique. Blood was subsequently allowed to clot at room temperature. Aliquots of the centrifuged samples were stored at −70 °C until evaluation.

### Biochemistry

Basic serum chemistry included evaluation of calcium, phosphate, creatinine, parathyroid hormone, 25-OH-vitamin D, and alkaline phosphatase. The bioactive form of sclerostin was measured with the quantitative sandwich ELISA kit from Biomedica (Bioactive Sclerostin ELISA BI 20472; Vienna, Austria). The intra-assay and interassay coefficients of variation (CVs) were < 1% and ≤ 5%, respectively. All samples were run in duplicate and in order to reduce inter-assay variation. They were analyzed in a single session. Bone turnover markers were evaluated as well. The bone resorption marker cross-linked-C-telopeptide of type I collagen (CTX; Cobas 8000 Roche Analyzer, Roche Diagnostics, Switzerland; detection limit: 0.5 ng/mL; intra-assay coefficient of variation: 1.2–4.7%, inter-assay coefficient of variation: 1.5–5.7%) as well as the bone formation markers osteocalcin (Oc; Cobas 8000 Analyzer, Roche Diagnostics, Switzerland; detection limit: 0.01 ng/mL; intra-assay coefficient of variation: 0.9–1.3%, inter-assay coefficient of variation: 1.2–2.3%) and *N*-terminal propeptide of type I collagen (P1NP; Cobas 8000 Roche Analyzer, Roche Diagnostics, Switzerland; detection limit: 5 ng/mL; intra-assay coefficient of variation: 1.6–3.5%; inter-assay coefficient of variation: 2.0–3.8%) were determined. All analyses were conducted according to standard procedures.

### Bone mineral density measurement

The BMD was measured at the lumbar spine and the proximal femur by dual energy x‑ray absorptiometry (DXA) using DXA devices of two manufacturers (Lunar Corp., Madison, WI, USA and Hologic, Waltham, MA, USA). To ensure high quality of the data cross-calibration was performed with the use of the European Spine Phantom.

### Statistical analysis

Continuous variables were described by median and quartiles due to non-normal distributions of most variables. Wilcoxon’s rank-sum test was used for comparison between sexes. Serum levels of bioactive sclerostin were log-transformed for centre-adjusted comparison between sexes using an ANCOVA model. The resulting mean difference was back-transformed to the original scale resulting in a percent increase with 95% confidence interval (CI). Correlations were quantified using a Spearman correlation coefficient partialized for center. Two-sided *p*-values below 0.05 were considered to indicate statistical significance. All calculations were done using the statistical analysis software SAS 9.4 (SAS Inc., 2016).

## Results

**Fig. 1:**
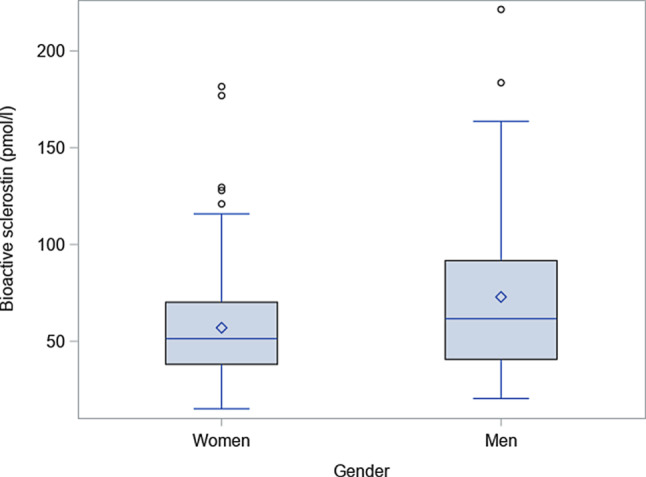
Distribution of serum levels of bioactive sclerostin by gender. Data are given as boxplots

**Fig. 2:**
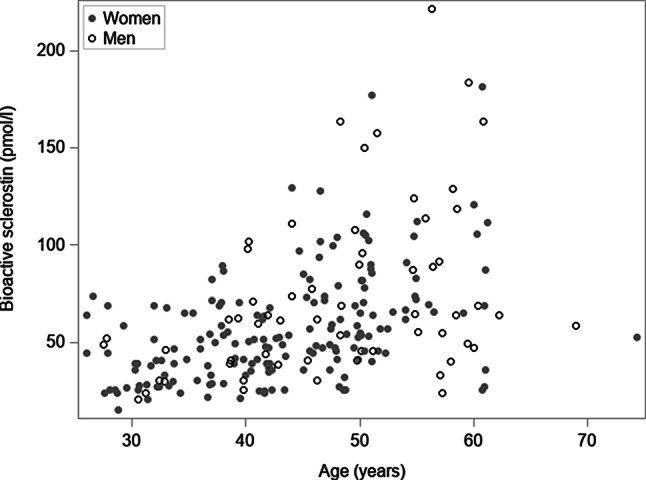
Scatterplot showing the relation of serum levels of bioactive sclerostin and age

Characteristics and biochemical parameters of 175 women and 61 men are given in Table 1. Men were older and had a higher BMI than women. The BMD and most routine clinical chemistry parameters were similar in both groups. Serum levels of bioactive sclerostin were 24% higher in men than in women (95% CI 9–41; *p* = 0.002; Fig. 1). The levels correlated positively with age (r = 0.47; *p* < 0.001; Fig. 2). As shown in Table 2, a weak positive correlation was also detected with BMI (r = 0.32; *p* < 0.001) as well as the T score of the femoral neck (r = 0.20; *p* = 0.002).

**Table 1:** Characteristics and biochemical parameters

	Women (*N* = 175)	Men (*N* = 61)	*p*
Age (years)	43 [37, 50]	49 [41; 56]	0.001
BMI	24 [21; 27]	26 [24; 28]	<0.001
T score lumbar spine	−0.4 [−1.0; 0.6]	0.0 [−0.6; 1.0]	0.053
T score femoral neck	−0.3 [−0.9; 0.4]	−0.2 [−0.7; 0.6]	0.189
Calcium (mmol/l)	2.4 [2.3; 2.5]	2.3 [2.2; 2.4]	0.011
Phosphate (mmol/l)	1.2 [1.1; 1.3]	1.2 [1.1; 1.4]	0.931
Alkaline phosphatase (U/l)	68 [54; 84]	74 [62; 84]	0.143
PTH (pg/ml)	28 [20; 38]	27 [19; 40]	0.981
25-OH-vitamin D (mmol/l)	16 [12; 25]	19 [14; 27]	0.090
Creatinine (mg/dl)	0.8 [0.8; 0.9]	1.0 [0.9; 1.2]	<0.001
Bioactive sclerostin (pmol/l)	51.3 [38.1; 70.2]	61.6 [40.6; 91.6]	0.002
CTX (ng/ml)	0.15 [0.09; 0.21]	0.17 [0.12; 0.27]	0.013
Osteocalcin (ng/ml)	20.9 [16.1; 26.2]	22.4 [19.2; 26.2]	0.100
P1NP (ng/ml)	36.7 [28.3; 47.7]	38.3 [30.5: 51.3]	0.391

Data are expressed as median [IQR]

*N* number of subjects, *BMI* body mass index, *PTH* parathyroid hormone, *CTX* cross-linked-C-telopeptide of type I collagen, *P1NP N*-terminal propeptide of type I collagen, *IQR* interquartile range

**Table 2:** Spearman correlation of bioactive sclerostin with age, body mass index and bone mineral density

	r	*p*
Age	0.47	<0.001
BMI	0.32	<0.001
T score lumbar spine	0.1	0.059
T score hip	0.2	0.002

*BMI* body mass index

Gender-specific correlation analyses corroborated the positive association of bioactive sclerostin with age (males: r = 0.38, *p* = 0.003; females: r = 0.47, *p* < 0.001). In men (r = 0.34; *p* < 0.01) as well as in women (r = 0.26; *p* < 0.001) a relatively weak correlation with BMI was found. Concerning the correlation with the T score of the femoral neck, the correlation coefficients were 0.23 (*p* = 0.07) and 0.15 (*p* < 0.05) in men and women, respectively. Additionally, a weak negative association between bioactive sclerostin and osteocalcin levels was detected in males (r = −0.29 *p* = 0.0289). No other associations between bioactive sclerostin and bone turnover markers could be established.

## Discussion

This is the first study evaluating serum levels of bioactive sclerostin in a population-based cohort. It revealed higher levels in men than in women and a positive association with age, body mass index, and bone mineral density for males and females. A correlation with the bone formation marker osteocalcin could only be observed in males.

This study’s results of higher levels in men than in women concur with the previous literature using different sclerostin ELISAs. Except for one study [11] which could not detect higher sclerostin levels in men after adjusting for several potential confounding factors, all other investigations [6–9, 19] found higher serum sclerostin levels in men than in women. That may be related to the following fact: osteocytes are the main source of sclerostin. Thus, circulating sclerostin levels may reflect the amount of osteocytes available. Of course, skeletal mass is larger in men than in women.

The age-associated increase of serum levels of bioactive sclerostin detected in this study is in line with most previous investigations, all of them using one of the former sclerostin ELISA assays. Except for an in vitro study which could not find age-related changes in sclerostin expression [20], most clinical studies detected an age-associated increase of serum levels of sclerostin [6, 11–14]; however, Dovjak et al. [7] and Moriwaki et al. [9] found an age-associated increase of serum sclerostin in men only. Study groups [10, 21, 22] that investigated solely postmenopausal women could not detect an association between serum sclerostin and age; however, this study revealed a positive association of bioactive sclerostin with age in men and women.

The positive correlation between serum levels of bioactive sclerostin and BMI is in accordance with publications by Amrein et al. [11] and Kalem et al. [22]. Several other studies [7, 10, 14, 19] could not detect such an association. With 26–74 years this study had a remarkably similar age range as Amrein et al. [11]. The other studies investigated either subjects of higher age or very young and older subjects. Differences in age may be responsible for the presence or absence of a positive correlation. No previous study performed a correlation analysis for women and men separately. This study confirmed the positive association between serum levels of bioactive sclerostin and BMI for men as well as for women.

Since sclerostin is predominantly produced by osteocytes several studies investigated potential associations between serum sclerostin levels and bone mass. Not all [12, 22] but most studies detected a positive correlation between serum sclerostin and BMD [6, 8, 10, 11, 23] and some also a correlation with better bone microarchitectural parameters or a lower fracture risk [6, 24]. This study’s low correlation is in line with Amrein et al. [11]. Studies with a higher level of correlation investigated subjects of higher age [8, 23]. Mödder et al. [6] divided their participants into different age categories and detected that the age group 60 years plus showed a higher correlation between SOST and BMD than subjects between 40 and 59 years of age, whereas in the even younger age group no correlation was found. Thus, this study’s correlation between serum levels of bioactive sclerostin and the T score of the femoral neck is in line with the previous literature. It is also in accordance with the finding that in osteoporotic bone characterized by a decrease of bone volume the expression of mRNA SOST is reduced [25]. So far only one study [6] performed a correlation analysis for both genders separately and found a positive correlation for men as well as for women above the age of 40 years for spine and total body BMD. In our study a positive association with the T score of the femoral neck was found in women and men; however, the level of significance could not be reached in men, a fact which most likely is due to the lower number of male participants.

In men, this study detected a weak negative correlation of bioactive sclerostin with osteocalcin (−0.29, *p* = 0.0289). Several other studies investigated the association of sclerostin with bone formation markers, mostly P1NP. All of them [6, 7, 9–11, 14, 26] but one [21] also revealed a negative association of the same dimension.

Participants’ vitamin D levels were well below the normal range; however, we do not believe that vitamin D deficiency influenced bioactive sclerostin levels. In kidney transplant recipients, a positive correlation between sclerostin and vitamin D has been found [27]. Nevertheless, in this study in line with a recent investigation of perimenopausal and postmenopausal women [28] no correlation between vitamin D and bioactive sclerostin levels could be detected. Median values of all bone turnover markers were within the normal range. Osteocalcin levels were similar to the study performed by Amrein et al. [11]. The fact that most women participating in this study were premenopausal may explain the relatively low CTX levels.

One limitation of this study is the cross-sectional design. Of course, it would have been even more informative to compare this bioactive sclerostin ELISA with different previous non-bioactive sclerostin ELISAs; however, limited sample volumes only permitted the determination of one instead of all sclerostin ELISAs. Thus, results of this study were compared with data of the previous literature using different sclerostin ELISAs.

## Conclusion

Higher serum levels of bioactive sclerostin in men than in women and the positive association with age shown in this population-based cohort study are consistent with data in the literature obtained by different sclerostin assays. Thus, according to this study it is possible to use a sclerostin ELISA as well as a bioactive sclerostin ELISA. Consequently, the determination of sclerostin concentrations in peripheral blood appears to be a robust parameter of bone metabolism.
